# Key terms for the assessment of the safety of vaccines in pregnancy: Results of a global consultative process to initiate harmonization of adverse event definitions

**DOI:** 10.1016/j.vaccine.2015.07.112

**Published:** 2015-09-19

**Authors:** Flor M. Munoz, Linda O. Eckert, Mark A. Katz, Philipp Lambach, Justin R. Ortiz, Jorgen Bauwens, Jan Bonhoeffer

**Affiliations:** a Departments of Pediatrics and Molecular Virology and Microbiology, Baylor College of Medicine, Houston, TX, USA; b Department of Obstetrics and Gynecology, University of Washington, Seattle, WA, USA; c Independent Consultant, Tel Aviv, Israel; d Initiative for Vaccine Research, World Health Organization, 20 Avenue Appia, 1211 Geneva 27, Switzerland; e Brighton Collaboration Foundation, Basel, Switzerland; f University of Basel Children’s Hospital, Basel, Switzerland

**Keywords:** Maternal immunization, Safety, Vaccines, Pregnancy, Brighton, AEFI

## Abstract

**Background:**

The variability of terms and definitions of Adverse Events Following Immunization (AEFI) represents a missed opportunity for optimal monitoring of safety of immunization in pregnancy. In 2014, the Brighton Collaboration Foundation and the World Health Organization (WHO) collaborated to address this gap.

**Methods:**

Two Brighton Collaboration interdisciplinary taskforces were formed. A landscape analysis included: (1) a systematic literature review of adverse event definitions used in vaccine studies during pregnancy; (2) a worldwide stakeholder survey of available terms and definitions; (3) and a series of taskforce meetings. Based on available evidence, taskforces proposed key terms and concept definitions to be refined, prioritized, and endorsed by a global expert consultation convened by WHO in Geneva, Switzerland in July 2014.

**Results:**

Using pre-specified criteria, 45 maternal and 62 fetal/neonatal events were prioritized, and key terms and concept definitions were endorsed. In addition recommendations to further improve safety monitoring of immunization in pregnancy programs were specified. This includes elaboration of disease concepts into standardized case definitions with sufficient applicability and positive predictive value to be of use for monitoring the safety of immunization in pregnancy globally, as well as the development of guidance, tools, and datasets in support of a globally concerted approach.

**Conclusions:**

There is a need to improve the safety monitoring of immunization in pregnancy programs. A consensus list of terms and concept definitions of key events for monitoring immunization in pregnancy is available. Immediate actions to further strengthen monitoring of immunization in pregnancy programs are identified and recommended.

## 1. Introduction

The concept of maternal immunization – vaccinating pregnant women in order to protect women themselves and their newborn infants from serious infectious diseases – emerged along with the development of the first vaccines in the early 20th Century [1]. Routine vaccination of pregnant women with tetanus toxoid has been successfully implemented worldwide since the 1960s for the prevention of maternal–neonatal tetanus [2]. In some countries, the recognition of severe influenza disease in pregnant women has led to the recommendation to vaccinate women with influenza vaccine [3,4]. The resurgence of pertussis disease in the United States and the United Kingdom has led those countries to recommend vaccination of pregnant women to prevent pertussis in infants [5,6]. Since the 1980s, the United States National Institutes of Health (NIH) has funded clinical studies of vaccines in pregnancy [7]. Worldwide, studies evaluating the safety, immunogenicity, and efficacy of various licensed and investigational vaccines in pregnancy against influenza, tetanus, *Haemophilus influenzae* type b, pneumococcus, meningococcus, group B streptococcus (GBS), *Bordetella pertussis* and respiratory syncytial virus (RSV) have been completed or are underway [7].

Although many studies and surveillance systems have collected information on reported adverse events following immunization (AEFI) in both mothers and their infants, there is variability in the terms and definitions of the events observed and assessed for a potential causal association. Since 2000, the Brighton Collaboration (BC), an independent professional network with the mission to enhance the science of vaccine research by providing standardized, validated objective methods for monitoring safety profiles and benefit–risk ratios of vaccines has provided investigators with case definitions of AEFI [8]. In 2004, the Brighton Collaboration was requested by WHO to develop a guidance document harmonizing safety assessment during maternal and neonatal vaccine trials. This document has been updated repeatedly in response to the rapidly evolving field [9]. In 2011, the NIH convened a series of meetings of experts with the goal of producing guidance to researchers in the field of maternal immunization, including recommendations concerning adverse events [10–14]. These NIH guidance documents were designed with high resource settings in mind, where research on maternal vaccines mostly had been conducted. Further attention to maternal immunization has been given by WHO which recently recommended that pregnant women receive influenza and pertussis vaccination under certain circumstances [15–17]. Highlighting the urgency and need for tools to standardize assessment of vaccine safety in pregnancy in all resource settings, large studies of vaccines for pregnant women, against influenza, pertussis, GBS, and RSV are now being planned or implemented in low- and middle-income countries [7,18,19].

No consensus vaccine safety monitoring guidelines or adverse event definitions to meet the need of concerted safety monitoring during the life cycle (development and post-licensure monitoring) of vaccines for global access in rapidly emerging immunization in pregnancy programs exist. This report describes the process pursued by BC and the WHO Initiative for Vaccine Research to advance the development of these necessary vaccine safety monitoring tools.

## 2. Methods

In 2014, Brighton Collaboration, together with WHO, convened two taskforces to conduct a landscape analysis of current practice, available terms, and case definitions and to develop and to propose interim terminology and concept definitions for the evaluation of the safety of vaccines administered to pregnant women. One taskforce reviewed maternal and obstetric events, and the other reviewed fetal and newborn events. Taskforce membership reflected diverse geographic and professional backgrounds, as well as broad expertise in clinical research, epidemiology, regulatory and immunization implementation requirements, maternal immunization, obstetrics, and pediatrics. Members represented academia, the pharmaceutical industry, regulatory agencies, clinical investigators, private and public organizations. The taskforces gathered relevant information from a systematic review of published literature on the safety of vaccination during pregnancy in mothers and infants as well as from a global stakeholder survey of relevant terms and safety assessment methods.

The objective of the systematic literature review was to determine the extent and variability in AEFI definitions and reporting in maternal immunization studies. The methods and results of the review were reported separately [20]. The objective of the global stakeholder survey was to identify existing case definitions of key events in pregnant women and newborns, as well as to describe existing methods for the assessment of safety of vaccines used in pregnancy. We developed an expansive list of national and international obstetric and pediatric professional societies, government agencies, regulatory agencies, research institutions, local and international organizations, and pharmaceutical companies that could be involved in work relevant to our objectives. We sent each institution an electronic survey and asked them to describe activities that collected information on key events during pregnancy and the newborn period. We also searched for information in existing standard terminology criteria documents, healthcare databases, population-based surveys, pregnancy registries, active and passive surveillance reporting systems, meeting and study reports, ongoing interventional and non-interventional studies, and the Brighton Collaboration network of vaccine safety experts. Through these efforts, we established an inventory of stakeholders and a repository of existing adverse event terms, case definitions, protocols, practice guidelines, and manuscripts with data pertinent to the assessment of safety of vaccines in pregnant women and their infants. The taskforces held regular meetings to define procedures, to review progress of information gathering, to prioritize event terms, and to recommend definitions of terms for further review at a larger expert consultation.

The taskforces identified “key terms”—defined as the most important adverse event terms based on frequency of occurrence, severity or seriousness, public health relevance, potential for public concern, and measurability or comparability with existing data. Key terms were organized with their synonyms (if pertinent), and existing definitions with bibliographic sources. When a taskforce identified more than one existing definition, it proposed a best definition based expert assessment of definition applicability and positive predictive value. These key terms, synonyms, and proposed definitions were presented at the expert consultation for further discussion.

The expert consultation took place at WHO in Geneva, Switzerland, July 24–25, 2014 and it included taskforce participants and other invited experts [21]. The objectives of the consultation were: (1) to review existing relevant obstetrical and pediatric adverse event case definitions and guidance documents; (2) to prioritize terms for key events for continuous monitoring of immunization safety in pregnancy; (3) to develop concept definitions for these events; and (4) to recommend a core data set of key terms of events to be collected when monitoring the safety of immunization in pregnancy. The terms and definitions were intended to be used specifically in vaccine safety monitoring. They were not intended to be used for diagnosis or treatment of patients, nor in non-vaccine clinical epidemiologic studies.

The taskforces proposed key terms and concept definitions. For each term, the full consultation determined whether the term was important for the assessment of safety of vaccines in pregnancy (i.e. was a “key term”), identified potential synonyms, determined whether there was consensus agreement on concept definitions, and considered the applicability of the term and concept definitions in different resource settings. This led to a list of key terms with synonyms and short descriptions of the respective disease concept, recognizing that this was a first critical step towards globally harmonized safety monitoring. It was acknowledged that an approach to reducing misclassification of reported events and to promoting data comparability in globally concerted safety monitoring would require more elaborate standardized case definitions. Such definitions should allow the classification of events based on objective, as well as measurable criteria at different levels of diagnostic certainty to serve the needs of monitoring the safety in diverse cultural and resource settings during the vaccine life cycle. Selected key terms were further classified as “priority outcomes” if they were considered to be the most important terms for the assessment of safety of the vaccine in pregnancy, “outcomes” if there were considered important but not critical, and “enabling” if the term was used to assist in the assessment of other outcomes or priority outcomes.

Overall, for organizational and reporting purposes, key terms were classified in broad conceptual categories. Key terms for the safety assessment related to immunization of pregnant women were sub-classified as: pregnancy-related, complications of pregnancy, complications of labor and delivery, and maternal health terms. Key terms for the assessment of safety in the fetus and newborn were sub-classified as: events of delivery, physical examination and anthropometric measurements, and neonatal complications classified by organ system.

## 3. Results

### 3.1. Systematic literature review of adverse event definitions

The results of the systematic literature review are reported in detail separately [20]. Briefly, among 74 studies included in the review, 10 were clinical trials, 54 were observational studies, and 10 were reviews. Most studies were related to influenza vaccine, followed by yellow fever vaccines, and then Tdap. A total of 240 different types of AEFI were reported on in these studies. Of these, 230 were systemic and 10 were injection site reactions. Considerable variability of the event terms used and lack of consensus on the definitions used for the assessment of AEFI reported in immunization in pregnancy studies was identified, rendering meaningful meta-analysis or comparison between studies and products challenging.

### 3.2. Stakeholder survey

**Fig. 1.**
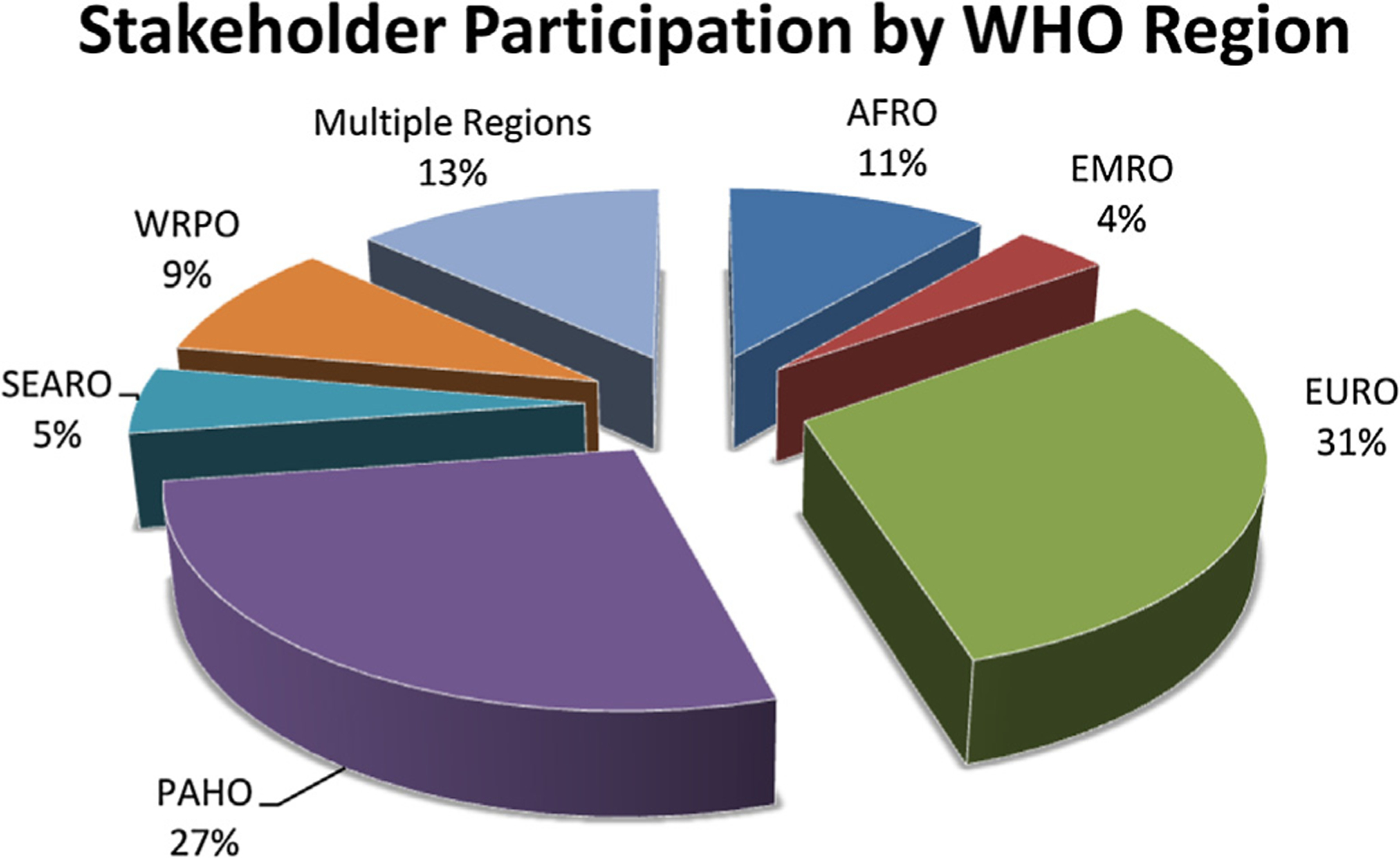
Stakeholder participation by WHO Region.

WHO contacted 446 individuals, and Brighton Collaboration contacted 500 individuals. Overall, 41% of individuals responded, and 40% of institutions responded. Individuals represented 427 institutions, of which 57% were based in the EURO and PAHO WHO regions. Of the institutions that responded, 81% were from the EURO and PAHO WHO regions (Fig. 1). Respondents confirmed the lack of standardized definitions for the assessment of safety of vaccines in pregnant women and reported their adverse event reporting to be based on classifications of events and terms used for various medical purposes or developed for use in a given organization of research network. Individuals shared the actual case definitions, protocols, and manuscripts used, when available. The survey identified relevant information from a wide variety of groups, including Brighton Collaboration documents on safety of vaccines, WHO documents addressing vaccine safety and surveillance of AEFI [9,22–26], the National Institutes of Health Toxicity Tables, publications and studies [10–14,27,28], the US National Children Study project [29–31], the Global Alliance on Prevention of Prematurity (GAPPS) [32], reports from GAVI Alliance, UNICEF and WHO [33–40], established terminology databases including the International Classification of Diseases (ICD-9, ICD-10) [41,42], Common Terminology Criteria for Adverse Events (CTCAE) [43], Medical Dictionary for Regulatory Activities (MedDRA) [44] and pregnancy and birth defect registries and guidance documents [45–55], vaccine safety and pharmacovigilance surveillance systems including the CIOMS report on vaccine pharmacovigilance [56], vaccine safety active surveillance programs [57–60], the American College of Obstetrics and Gynecology (ACOG) practice guidelines [61,62], investigators of current and planned clinical trials, and the pharmaceutical industry working on candidate vaccines for pregnant women.

### 3.3. Key terms and consensus definitions

Based on the findings of the landscape analysis and predefined criteria as described above, a total of 45 key terms describing medical events of significance for the assessment of safety of vaccines in pregnant women were identified by the maternal and obstetric event taskforce. A total of 62 key terms were identified by the neonatal and fetal event taskforce. The participants of the consultation recommended the elaboration of disease concepts into standardized case definitions with sufficient applicability and positive predictive value to be of use for monitoring the safety of immunization in pregnancy globally. Overall, 39 key terms were reviewed, prioritized and agreed upon by the participants of the consultation and consensus concept definitions were endorsed for immediate use. A summary of all key terms are described in Tables 1 and 2, respectively. A complete repository including additional suggested terms and interim concept definitions suggested by the taskforces is available at the Brighton Collaboration website [63].

### 3.4. Other recommendations

In addition, the expert consultation identified and recommended critical steps to further improve safety monitoring of immunization in pregnancy programs (Table 3), including the development of guidance for data collection, analysis and presentation of safety data; tools for harmonized data collection, classification, and data sharing; and globally concerted secondary use of health care datasets to strengthen active surveillance to enable evidence based local and global response to safety concerns [20].

## 4. Discussion

We conducted a literature review, global stakeholder survey, and expert consultation to assess key events related to safety monitoring of immunization in pregnancy. We identified substantial heterogeneity of event definitions and assessment methods in current practice, and described a structured approach to initiating globally concerted action towards the ascertainment of the safety of mothers and their children following immunization in pregnancy.

The systematic literature review was a hallmark of this consensus process, highlighting the opportunities for improvement. The strengths and limitations of this effort are discussed in detail elsewhere [20]. The findings directly informed decision making and prioritization both at the taskforce and consultancy levels, and provide a useful baseline assessment for monitoring and re-evaluation of globally concerted actions in this rapidly evolving field of research.

The stakeholder survey was the second hallmark of consensus formation. Given the thorough approach, we interpret the response rate and geographic distribution of responses to be reflective of the actual availability of event terminologies, case definitions, and guidance documents in the regions where most of the structured research into the safety of drugs and vaccines administered during pregnancy have thus far been conducted. The expert consultation recommended efforts be made to increase involvement from low- and middle-income countries, particularly in Africa and Asia, as trials and immunization programs are increasingly occurring in these regions.

WHO and the Brighton Collaboration continue to monitor emerging case definitions and guidance documents, as well as validation efforts informing best practice and harmonization efforts for upcoming vaccines and programs of immunization in pregnancy. The authors recognize that despite the taskforces’ efforts to capture existing definitions for key safety events in pregnancy, it is likely we have not identified all definitions available or needed. Thus, we encourage readers to share available information not captured or adequately represented in this publication by contacting the WHO (VaccineResearch@who.int) and Brighton Collaboration (contact@brightoncollaboration.org).

While challenging, the development of a common language through harmonized definitions will facilitate efforts in the research and implementation of vaccines for maternal immunization. The consistent use of definitions of key events related to immunization in pregnancy will enhance comparability of safety outcomes monitored during the vaccine life cycle from pre-licensure to post-licensure clinical trials, as well as from observational studies.

Harmonization of terms, disease concepts and the development of standardized case definitions of key events related to safety monitoring of immunization in pregnancy is a challenging exercise, specifically in view of the need for applicability in high- and low-income settings and the multiple stakeholders involved. We employed a structured approach building on the recognized standard Brighton Collaboration process [8] to arrive at interim terminology and concept definitions for immediate use, while planning for collaborative development and validation of standardized case definitions with investigators and stakeholders in the near future. We recognize that the establishment of a core set of terms, disease concepts, and definitions is an important step towards this aim, while acknowledging that not all pertinent events may be identified and defined in anticipation. However, with an established network and processes among globally collaborating investigators, additional ad hoc definitions may be developed rapidly as the need arises.

Therefore, an important aspect of this effort was the broad net that was cast to identify relevant methods, terms, and definitions available from all resource settings. The early involvement and contributions by a large group of stakeholders with diverse backgrounds and the global expertise within the taskforces and in the consultation strengthened the harmonization process from its inception. Broad representation and face-to-face discussion encouraged increasing information exchange and collaboration, while minimizing duplication of efforts.

The harmonization exercise and consultancy also helped foster discussion on the necessary way forward given current limitations. The participants identified additional obstacles and needs. Recommendations included the development of tools to standardize and increase the efficiency of safety data collection in clinical trials and observational studies. Further, robust data on background rates of key events related to immunization in pregnancy, and pooled safety analyses based on international data sharing would better inform decision making on maternal immunization programs, and enhance patient, regulator, and provider decision making and comfort with vaccination offered to protect pregnant women and their children from preventable diseases and possible death.

Maternal immunization is an evolving field, and adaptation of standards and tools to specific vaccines, protocols, populations, geographic regions, and other factors is necessary when evaluating the safety of vaccines in pregnancy. The Brighton Collaboration has established a collaborative network dedicated to address this continuing need: the Global Alignment of Immunisation Safety Assessment in Pregnancy (GAIA) [63,64]. The aim of the GAIA project is to provide standards and tools to establish a globally shared understanding of outcomes and approaches to monitoring them with specific focus on low- and middle-income countries needs and requirements. GAIA will build on the efforts of this initial work and develop standardized case definitions for selected key terms through the standard Brighton process as well as guidance and tools harmonizing data collection in clinical trials and observational studies.

The process described in this paper outlines a format successfully initiating active discussion and sharing of information between stakeholders and investigators in view of rapidly evolving immunization programs of pregnant women. This approach could serve as a model for future efforts aiming at early harmonization of the safety assessment of specific vaccines and global immunization programs leading to sustainable collaboration and concerted action while minimizing fragmentation and duplication of efforts in line with the Global Vaccine Safety Blueprint, the strategic plan of the WHO Global Vaccine Safety Initiative [65].

## 5. Disclaimer

Philipp Lambach and Justin Ortiz work for the World Health Organization. The authors alone are responsible for the views expressed in this publication. The findings, opinions, and assertions contained in this document are those of the individual scientific professional contributors. They do not necessarily represent the official positions of each contributor’s organization (e.g., government, university, or corporation). Specifically, the findings and conclusions in this paper are those of the authors and do not necessarily represent the views of the author’s organizations.

## Figures and Tables

**Table 1:** Maternal key terms and concept definitions.

Term/synonyms	Concept definition	Prioritization
Pregnancy related terms		
Pregnancy	Period from when the woman misses her last normal menstrual period (first day of last menstrual period plus two weeks), to the onset of labor/elective caesarian section or abortion (WHO)	Taskforce key term
Diagnosis of pregnancy	Absence of menses combined with ultrasound documentation of pregnancy or physical examination documenting enlarged uterus with detection of fetal heart beat	Taskforce key term
	Note: Absence of menstruation with confirmation by urine or serum test for presence of Beta-human chorionic gonadotropin (B-HCG) is presumptive evidence for pregnancy	
Gestational age estimate* S: Dating of Pregnancy Duration of pregnancy	Dating of pregnancy from the first day of the last menstrual period (LMP) with confirmation by (in order of most to least accurate dating):	Enabling term
	1st trimester ultrasound	
	1st trimester examination consistent with LMP	
	detection of fetal heart beat consistent with LMP and uterine size	
	2nd trimester ultrasound	
	3rd trimester ultrasound	
	Examination of the fetus	
	OR	
	Known date of fertilization (e.g. Assisted Reproductive Technology (ART), Intrauterine Insemination (IUI))	
Trimesters	Divisions of pregnancy into 3 parts that roughly correspond with maternal physiologic and fetal development phases.	Taskforce key term
	1. NCS/NICHD divisions	
	1st: ≤12 weeks	
	2nd: 13–26 weeks from LMP	
	3rd: 27–40 weeks from LMP	
	2. WHO divisions	
	1st: <4 mo or <16 weeks	
	2nd: 4–6 mo or 16–28 weeks	
	3rd: 7–9 mo or 29–40 weeks	
Term pregnancy*	A gestation of 37–41 6/7 weeks or 259 to 294 days in duration (NCS)	Outcome
Antenatal care	Care for the woman and fetus during pregnancy (WHO)	Taskforce key term
Post-partum care	Care of the woman from delivery of placenta to 42 days after delivery (WHO)	Taskforce key term
Peri-partum period	Interval between delivery after 20 weeks gestation and 28 days after delivery	Taskforce key term
Puerperium	4–6 weeks period of time following delivery	Taskforce key term
Complications of pregnancy		
Abortion* S: Pregnancy Loss Miscarriage	Pregnancy loss before 22 weeks of gestation	Outcome
	Note: Definition of fetal viability varies in different resource settings: 20–22 weeks in high resource versus 28 weeks in LMIC resource settings and corresponding fetal weight of 500 g vs 1000 g	
	Categories	
	Spontaneous: Pregnancy loss happens without any preceding intervention	
	Elective: Intervention (medicine or procedure) leads to pregnancy loss	
	Early: ≤12 weeks (ACOG) or <14 weeks (WHO)	
	Late: 13–22 weeks	
	Complete: All products of conception pass from uterus	
	Incomplete: Products of conception remain in uterus (Retained products of conception)	
	Threatened: First trimester bleeding	
In utero fetal demise (IUFD)	Death of a fetus in utero	Taskforce key term
	Early fetal death: <14 weeks	
	Late fetal death: 14 to <20 or 22 weeks	
Labor*	Regular uterine contractions AND cervical change. Changes in the cervix include effacement (thinning) and dilation (opening).	Enabling term
Preterm Labor* S: premature labor	Labor prior to 37 weeks gestation	Priority outcome
	Note: Contractions without cervical change is NOT preterm labor	
Pre-eclampsia* S: Toxemia HTN in pregnancy	A pregnancy-related disorder characterized by an increase in the blood pressure after the 20th week of gestation, and up to 6 weeks post-partum, combined with other abnormalities. (NCS)	Priority outcome
	Blood pressure ≥140 systolic or ≥90 diastolic on two occasions at least 4 h apart after 20 weeks of gestation in a woman with previously normal blood pressures and protein in urine, OR Blood pressure ≥160 systolic or ≥110 diastolic twice in a short time interval	
	PLUS	
	Proteinuria >300 mg of protein in 24 h (or this amount extrapolated from a timed collection) OR Protein/Creatinine ratio > 0.3 OR 1+ in urine dip	
	OR	
	Elevated BP with onset of any of: Platelets <100,000, Serum Creatinine >1.1 OR a doubling of serum Creatinine, OR Liver transaminases twice normal (ACOG)	
Pre-eclampsia with severe features* S: Severe preeclampsia Severe toxemia	Pregnancy related disorder of severe hypertension and/or some other abnormalities (NCS)	Priority outcome
	Pre-eclampsia associated with any of the following findings: (ACOG)	
	(1) thrombocytopenia (platelets less than 100,000 per microliter)	
	(2) impaired liver function	
	(3) twice normal elevation of hepatic transaminases	
	(4) severe, persistent right upper quadrant or epigastric pain)	
	(5) progressive renal insufficiency (serum creatinine greater than 1.1 mg/dL or doubling of baseline in the absence of other renal disease)	
	(6) pulmonary edema	
	(7) new-onset cerebral or visual disturbances	
Eclampsia* S: seizure of pregnancy	In a woman with pre-eclampsia, a convulsion that cannot be attributed to another cause. (NCS)	Priority outcome
HELLP Syndrome	Variant of pre-eclampsia/eclampsia characterized by hemolytic anemia, elevated liver enzymes and low platelet count	Taskforce key term
Gestational hypertension* S: Pregnancy associated or induced hypertension (PIH)	Blood pressure ≥140 systolic/≥90 diastolic that starts after 20 weeks of gestation (measured twice at least 20 min apart AND absence of protein or other stigmata of preeclampsia)	Priority outcome
Chronic Hypertension with superimposed pre-eclampsia*	Pre-eclampsia in a woman with diagnosis of chronic hypertension	Priority outcome
Hyperemesis gravidarum	Severe, intractable vomiting during early pregnancy accompanied by hypovolemia, weight loss, and electrolyte imbalances	Taskforce key term
Fetal growth restriction* S: IUGR	A fetus that does not grow beyond the 10th% of conventionally accepted size for gestational age (NCS) Other reported criteria: fundal height 3 cm below the expected height between 24–38 weeks of gestation; ultrasound measured abdominal circumference (AC) <10%, Estimated fetal weight (EFW) <10%, EFW <10% with abnormal Doppler studies, or birthweight <2500	Priority outcome
Oligohydramnios	Low amniotic fluid in utero	Taskforce key term
	Ultrasound diagnosis: Total amniotic fluid index of ≤5 cm or single pocked ≤2 cm OR Total amniotic fluid <8 cm, or smallest vertical pocket <2 cm	
Gestational Diabetes Mellitus* S: Diabetes of pregnancy	Women with carbohydrate intolerance with onset during pregnancy	Suggested outcome
	Diagnosis based on administration of glucose challenge test at 24–28 weeks gestation	
Antenatal Bleeding*	Vaginal bleeding during pregnancy. Different etiologies and consequences depending on trimester of gestation	Suggested outcome
Placental Abruption* S: Abruption	Partial or total placental detachment after 20 weeks gestation prior to delivery of the fetus	Taskforce key term
		AND
		Suggested outcome
Complications of labor and delivery		
Pre-labor rupture of membranes (PROM)*	Spontaneous ruptured membranes at or after 37 weeks of gestation before the onset of labor	Outcome
Preterm pre-labor rupture of membranes (PPROM)*	Spontaneous ruptured membranes prior to <37 weeks of gestation before the onset of labor	Priority outcome
Duration of rupture of membranes	Time interval between rupture of membranes and birth 12 h is considered “prolonged” by some	Taskforce key term
Preterm delivery*	Delivery before 37 weeks of gestation are completed (WHO/CDC)	Outcome
	Subgroups (WHO):	
	Moderate to late Preterm: 32 to <37 weeks	
	Very Preterm: 28 to <32 weeks	
	Extreme Preterm: <28 weeks (included under Very Preterm category in some WHO definitions)	
Cesarean section (C/S) delivery* S: abdominal delivery	Delivery of fetus via abdominal incision (laparotomy) and then uterine incision (hysterotomy)	Enabling term
Fetal distress*	The presence of signs in a pregnant woman, before or during childbirth, that suggest that the fetus may not be well	Suggested outcome
	1. If electronic monitoring available:	Note: This is a complex term that needs further discussion and refinement
	Persistent category 2 tracing that does not improve with intervention or a category 3 tracing	
	2. If only spot monitoring with fetoscope or Doppler available:	
	Fetal bradycardia or tachycardia after a minimum of 2 min baseline assessment	
Dysfunctional labor S: Labor dystocia Failure to progress Arrest of dilatation	Prolonged time between labor beginning and delivery	Taskforce key term
	OR	Note: This is a complex term that needs further discussion and refinement
	Uterine contractions (less than 3 in 10 min or inadequate strength) that do not result in progressive cervical dilation	
FIRST stage labor dysfunction:	*Prolonged Latent Phase of Labor* (S: latent phase arrest; arrest of labor in the latent phase)	Taskforce key term
	Prolonged time before reaching active phase of labor	Note: This is a complex term that needs further discussion and refinement
	Greater than 20 h in nulliparous women and greater than 24 h in parous women	
	*Arrest of labor in the active phase* (S: prolonged active phase of labor; prolonged first stage of labor; active phase arrest)	
	Prolonged time between reaching active phase of labor and 2nd stage of labor ≥6 cm dilation with membrane rupture and one of the following: 4 h or more of adequate contractions (e.g. >200 Montevideo units), ≥6 h of inadequate contractions and no cervical change	
Second stage labor dysfunction: S: cephalopelvic disproportion (CPD)	Prolonged time between complete dilation and delivery of the fetus (2nd stage)	Taskforce key term
	For Nullipara: >4 h with epidural, or >3 h without epidural	Note: This is a complex term that needs further discussion and refinement
	For Multipara: >2 h with epidural and >1 h without epidural	
	*Arrest of descent*	
	After complete dilatation, failure of the fetal presenting part to descend through the pelvis	
Chorioamnionitis*	Inflammation of membranes around the fetus	Suggested outcome
	Inflammation of the fetal sac membranes, characterized by otherwise unexplained maternal fever (at or above 38 degrees C (100.4 F) with one of more of the following: uterine tenderness and/or irritability, leukocytosis, fetal tachycardia, maternal tachycardia, or malodorous vaginal discharge. (NCS)	
Post-partum endometritis S: Puerperal endometritis or endomyometritis	Infection of the uterus in the postpartum period	Taskforce key term
	Infection of the endometrium, decidua and/or myometrium occurring at any time between birth and 42 days postpartum. (NCS)	
Maternal Fever*	Elevation of body temperature ≥38°C	Outcome
Maternal sepsis S: Septicemia	Systemic inflammatory response to blood borne bacteria or viruses or their byproducts.	Taskforce key term
	Clinical syndrome defined by the presence of both infection and a systematic inflammatory response. (NCS)	
Post-partum hemorrhage*	Blood loss accompanied by signs and symptoms of hypovolemia in the first 24 h following the birth process	Priority outcome
Maternal death*	Death of a woman while pregnant or within 42 days of termination of pregnancy, irrespective of the duration and site of the pregnancy, from any cause related to or aggravated by the pregnancy or its management, but not from accidental or incidental causes	Priority outcome
	*Direct obstetric death*: death of the mother resulting from conditions or complications which are unique to pregnancy and occur during the antepartum, intrapartum, or postpartum period.	
	*Indirect obstetric death*: A maternal death that is not directly due to obstetric cause (such as from previously existing disease, or disease developing during pregnancy, labor, or the puerperium but that was not unique to pregnancy.)	
	*Late Maternal Death*: Death of woman from direct or indirect causes more than 42 days but less than one year after termination of pregnancy	
Maternal health terms		
Deep Vein Thrombosis (DVT)	A blood clot (thrombus) in a deep vein, predominantly in the lower extremity but may include the pelvis or upper extremity. (NCS)	Suggested outcome
HIV infection	HIV detected by accepted test	Taskforce key term and suggested enabling term
	Use WHO definitions of HIV www.who.int/topics/hiv_aids	
Nutritional Status	Pre-pregnant weight of mother	Taskforce key term
	Obesity: Body mass index (BMI) ≥ 30	
	Underweight (lack of proper nutrition): BMI < 18.5	
Maternal chronic hypertension*	Blood pressure > 140/90 before 20 weeks gestation or prior to pregnancy	Enabling term
Other maternal health	Anemia during pregnancy, purpura, maternal cardiomyopathy, maternal seizures, maternal neurologic disorders, autoimmune disorders	Suggested outcomes
Other post-partum events	Lactation, mastitis, uterine rupture	Suggested outcomes

Sources of Accepted Definitions: WHO: World Health Organization; NCS: National Children’s Study; NICHD: National Institute of Child Health and Human Development; ACOG: American Congress of Obstetrics and Gynecology; CDC: Center for Disease Control and Prevention.

* BC-WHO Consultation consensus term.

**Table 2:** Neonatal key terms and concept definitions.

Term/synonyms	Concept definition	Prioritization
Events of delivery		
Live Birth* S: Live born	Delivery of an infant, regardless of maturity or birth weight, as determined by the presence of a heartbeat or spontaneous respirations or spontaneous movement	Enabling term or outcome
Stillbirth* S: Stillborn Fetal Demise/Death Deadborn	Delivery of a dead fetus after 22 weeks of gestation (WHO)	Priority outcome
	Categories:	
	- Early Stillbirth Delivery ≥22 weeks and/or >500g	
	- Late Stillbirth Delivery ≥28 weeks and/or >1000g	
	Other commonly reported subgroups:	
	- Antepartum or during pregnancy or “macerated”	
	- Intrapartum, defined as no signs of life at delivery and more than 500 g or >22 weeks of gestation, with intact skin and no signs of disintegration in utero. The death is assumed to have occurred in the 12 h before delivery and to be more likely due to an intrapartum event. Excludes babies with severe congenital anomalies	
Perinatal death*	Death of fetus at or after 22 weeks of gestation and/or neonate up to 1 week (7 days) after birth	Priority outcome
Neonatal death*	Death of a live newborn at any time from birth to 28 days of life, regardless of gestational age.	Priority outcome
	Subgroups:	
	Very early neonatal death: <24 h	
	Early neonatal death: birth to <7 days	
	Late neonatal death: 7 to <28 days	
Infant death*	Death of a live born occurring from birth until 12 months of age	Priority outcome
	Subgroup: post-neonatal death: occurs between 28 days and 1 year of life	
Term birth*	Birth at ≥37 weeks to <42 weeks of gestation	Enabling term or outcome
Preterm birth*	Birth before 37 weeks of gestation are completed	Priority outcome
	Subgroups: (WHO)	
	Moderate to late Preterm: 32 to <37 weeks	
	Very Preterm: 28 to <32 weeks	
	Extreme Preterm: <28 weeks	
Early term birth*	Birth at 37 to <39 weeks of gestation	Outcome
Post-term birth* S: Post-mature birth	Birth on or after 42 weeks of gestation	Outcome
Physical examination and anthropometric measurements		
Low Apgar Scores*	Score of less than 7 on a 10 point Apgar scale after 5 min	Outcome
Low Birth Weight*	Birth weight below the normal birth weight range of 2500 to 3999 g	Priority outcome
	Subgroups:	
	Low birth weight (LBW): <2500 g	
	Very low birth weight (VLBW): <1500 g	
	Extremely low birth weight (ELBW):<1000 g	
High birth weight* S: Macrosomia	Birth weight ≥4000 g	Outcome
Small for Gestational Age (SGA)* S: IUGR = intrauterine growth restriction	Birth weight <10% for infants of same gestational age and gender in same population	Priority outcome
Large for Gestational Age (LGA)*	Birth weight >90% for infants of same gestational age in same population	Outcome
Birth length*	Crown-foot length in cm assessed in relation to gestational age	Enabling term
Microcephaly*	Head circumference >2 Standard deviations below mean for gestational age, gender and ethnic origin	Outcome
Macrocephaly*	Head circumference >2 standard deviations above mean for gestational age, gender and ethnic origin	Outcome
Congenital anomalies* S: Birth defects Malformations	Abnormalities of body structure or function that are present at birth and are of prenatal origin. (WHO)	Priority outcome
	MAJOR ANOMALIES	
	Those that require surgical/medical treatment, have serious adverse effects on health or development (functional), or have significant cosmetic impact.	
	MINOR ANOMALIES	
	Those that do not in themselves have serious medical, functional or cosmetic consequences for the child. Includes those found in association with major anomalies	
Neonatal conditions classified by organ system		
Systemic conditions		
Asphyxia*	Insufficient oxygen supply to organs at birth resulting from inadequate ventilation or perfusion	Priority outcome
Fever*	Elevated body temperature at or above 38 °C measured at least once (BC)^a^	Outcome
Hypothermia	Decreased body temperature below 36 °C	Taskforce key term
Infection*	Infection regardless if acquired in utero, intrapartum or in neonatal period	Priority outcome
	Congenital infection: Acquired in utero at any time of gestation and prior to delivery	
	Key Event: Infection caused by organism for which mother received vaccination during pregnancy (vaccine associated or vaccine failure)	
Sepsis	Infection associated with cardiovascular collapse and systemic, multiorgan involvement.	Taskforce key term
	Neonatal sepsis: Sepsis diagnosed in the first 28 days of life.	
	Accepted Categories:	
	(WHO)	
	Early onset: <7 days of age	
	Late onset: 7–90 days of age	
	(NCS) Early onset: <72 h of life	
	Late onset: ≥72 h of life	
Sudden infant death syndrome* S: SIDS, cot death	Sudden death of any child under 12 months of age which remains unexplained after excluding other causes of death (BC)^b^	Priority outcome
Failure to thrive or growth deficiency*	Inability to maintain expected growth rate over time, evaluated by plotting individual weight gain and growth on standard growth charts for the population	Suggested outcome and taskforce key term
Respiratory tract		
Respiratory distress*	Increase in respiratory rate above normal range for age and labored breathing (nasal flaring, grunting, retractions, pallor and cyanosis or hypoxemia). May be transient or persistent	Suggested outcome
Transient tachypnea of newborn S: TTN	Respiratory distress beginning shortly after birth and usually resolving over 24–48 h or within 3 days of delivery.	Taskforce key term
	Usually associated with retained lung fluid after delivery AND Cesarean section delivery with or without labor in term or preterm infants usually >35 weeks of gestation	
Meconium Aspiration syndrome S: MAS Aspiration pneumonia	Respiratory distress syndrome associated with presence of meconium stained amniotic fluid in the lungs during or before delivery.	Taskforce key term
	Usually associated with fetal distress prior to and at the time of delivery in term or post-term infants AND visual inspection of trachea and larger airways by endotracheal intubation and suctioning to determine the presence of meconium stained amniotic fluid	
Respiratory distress syndrome S: RDS Hyaline membrane disease Surfactant deficiency syndrome	A respiratory syndrome in premature infants caused by developmental insufficiency of surfactant production and structural immaturity in the lungs. Begins shortly after birth and is manifest by respiratory distress	Taskforce key term
Persistent pulmonary hypertension of the newborn S: PPHN Persistent fetal circulation	Persistence of fetal circulatory pattern of right to left shunting through the patent ductus arteriosus and foramen ovale after birth due to excessively high pulmonary vascular resistance. Begins shortly after birth, usually within the first 12 h of life, and is manifest by respiratory distress AND hypoxemia that is unresponsive to 100% oxygen and out of proportion with findings in chest X-ray. Usually occurs in term infants	Taskforce key term
Apnea*	Cessation of breathing for 15 (or 20) s or more, or a shorter respiratory pause associated with bradycardia, cyanosis or hypoxemia, pallor, and/or hypotonia. Should be distinguished from periodic breathing	Suggested outcome
Pneumonia	An inflammatory condition of the lung affecting primarily the alveoli. It is usually caused by infection with viruses or bacteria.	Taskforce key term
	Key event: Infection caused by organism for which mother received vaccination during pregnancy	
Chronic lung disease S: Bronchopulmonary dysplasia (BPD)	A chronic lung disorder characterized by inflammation and scarring in the lungs that is most common among infants who were born prematurely and result in need for supplemental oxygen	Taskforce key term
Neurologic/neuromuscular		
Neonatal Hypoxia* S: Neonatal Asphyxia	Decreased arterial concentration of oxygen and insufficient blood flow to cells or organs to maintain their normal function, particularly the central nervous system.	Taskforce key Term and Suggested outcome
	Related terms:	
	Asphyxia = insufficient oxygen supply to organs due to poor ventilation or poor perfusion	
	Anoxia = complete lack of oxygen	
	Hypoxia = decreased arterial concentration of oxygen	
	Ischemia = insufficient blood flow to maintain normal organ function	
Neonatal Encephalopathy* S: Birth Asphyxia Perinatal Asphyxia	Injury to the central nervous system that occurs when there is insufficient delivery of oxygen or blood to all or part of the brain (NCS)	Priority outcome and Taskforce key term
	OR	
	A disturbance of neurological function manifested by difficulty initiation and maintaining respiration, depression of tone and reflexes, abnormal level of consciousness and often seizures.	
	1. Due to intrapartum hypoxic insult	
	2. Due to another cause	
	May be mild, moderate or severe.	
	Assessed by clinical and laboratory findings including: 5 min Apgar score of 0–3; Respiratory distress and Acidosis (pH < 7.0); Altered tone, depressed level of consciousness, seizures; Multiorgan involvement; Abnormal CNS imaging or EEG. May result in neonatal death or permanent damage to the brain and other organs. May be associated with perinatal events, rarely to prenatal events	
Hypoxic Ischemic Encephalopathy* S: HIE	A syndrome of abnormal neurological behavior in the neonate, which is frequently associated with multi-system dysfunction and follows severe injury before or during delivery, associated with hypoxic and/or ischemic event.	Priority outcome and Taskforce key term
	May be mild, moderate or severe.	
	Comment: The term Neonatal Encephalopathy, specifying if it is associated with intrapartum event, is preferred	
Lethargy	Reduced responsiveness to environmental stimuli	Taskforce key term
Irritability*	Abnormal responsiveness to stimuli or physiologic arousal, may be in response to pain, fright, a drug, emotional situation or a medical condition (CTCAE)	Suggested outcome
Seizure* S: Convulsion	Witnessed sudden loss of consciousness AND generalized, tonic, clonic, tonic–clonic, or atonic motor manifestations (BC)^c^	Suggested outcome
Hypotonia/hypertonia	Decreased or increased muscular tone for gestational and post-natal age	Taskforce key term
Hyporreflexia/hyperreflexia	Decreased or increased reflexes for gestational and post-natal age	Taskforce key term
Meningitis	Inflammatory process of the meninges (BC)^d^	Taskforce key term
Meningoencephalitis	Inflammatory process of the meninges and brain parenchyma (BC)^e^	Taskforce key term
Intracranial intraventricular hemorrhage S: IVH	Bleeding in the ventricles or brain parenchyma	Taskforce key term
	Associated with prematurity or other factors such as trauma, RDS, hypoxia–ischaemia, hypo- or hypertension, other maternal and fetal factors.	
Periventricular leucomalacia S: PVL	Decreased perfusion, periventricular hemorrhage and/or necrosis in the periventricular white matter and/or white matter, Associated with prematurity and IVH, hypoxia-ischemia, other maternal and fetal factors	Taskforce key term
Sleeping issues*	Disturbance in sleep pattern	Suggested outcome
Neurodevelopmental disability*	Alteration in progression or regression of normal development of motor, speech or cognitive skills as expected for gestational and post-natal age	Suggested outcome
	Assessed by medical history, physical examination, and standard screening and assessment tools appropriate for age. Serial assessment required due to variability in individual acquisition of skills. Assessment at or beyond 1 year of age more likely to represent true disability	
Cardiovascular		
Tachycardia/Bradycardia*	Heart rate above or below normal range for age and gestational age	Suggested enabling term
Hypertension/Hypotension	Blood pressure above or below normal range for age, gestational age, gender and length and height	Taskforce key term
Heart failure	Cardiac dysfunction resulting in symptoms	Taskforce key term
Hematologic	
Bleeding*	Loss of blood from any site or etiology	Suggested outcome
	Assessed by Evidence of bleeding AND symptoms that may include tachycardia, hypotension, diaphoresis, lethargy, pallor, cyanosis, shock AND Anemia (low hemoglobin or hematocrit)	
Anemia*	Hematocrit or hemoglobin concentration below the lower limit of normal range for gestational age and post-natal age	Suggested outcome
Polycythemia	Hematocrit or hemoglobin concentration above the upper limit of normal range for gestational age and post-natal age	Taskforce key term
Thrombocytopenia*	Platelet count below the lower limit of normal range for gestational age and post-natal age (BC)^f^	Suggested outcome
Leukopenia	Decreased white blood cell count below lower limit of normal range for gestational and post-natal age	Taskforce key term
Leukocytosis*	Increased white blood cell count above upper limit of normal range for gestational and post-natal age	Suggested outcome
Coagulopathy S: DIC Disseminated intravascular coagulation	Bleeding and/or clotting disorder associated with abnormal activation of coagulation pathways	Taskforce key term
Gastrointestinal		
Difficulty feeding*	Poor suck and/or inability to maintain adequate oral intake for age	Suggested outcome
Vomiting	Reflexive act of ejecting stomach contents through the mouth	Taskforce key term
Diarrhea	Increase in frequency and/or change in consistency (liquid) of stools for age (BC)^g^	Taskforce key term
Necrotizing enterocolitis	Disease of the gastrointestinal tract characterized by mucosal or transmural necrosis of the intestine.	Taskforce key term
Jaundice/Hyperbilirubinemia*	Elevation of Total and/or Direct–Indirect bilirubin for gestational and post-natal age Subgroups:	Suggested outcome
	Physiologic hyperbilirubinemia	
	Neonatal hyperbilirubinemia	
	Direct/Conjugated hyperbilirubinemia	
	Indirect/Unconjugated hyperbilirubinemia	
	Breastfeeding Jaundice	
Hepatic dysfunction	Elevation of liver enzymes and/or coagulopathy	Taskforce key term
Metabolic		
Hypoglycemia	Low serum glucose concentration (mg/dL) below lower limit of normal for gestational and post-natal age	Taskforce key term
Hypocalcemia/Hypercalcemia	Concentration of serum (mg/dL) or ionized Calcium below or above the lower and upper limits of normal for gestational and post-natal age	Taskforce key term
Hypomagnesemia/Hypermagnesemia	Concentration of serum (meq/L) magnesium below or above the lower and upper limits of normal for gestational and post-natal age	Taskforce key term
Renal		
Renal insufficiency	Decreased urinary output and/or elevation of serum creatinine above upper limit of normal for gestational and post-natal age	Taskforce key term
Renal failure	Persistent oliguria or anuria with decreased creatinine clearance for gestational and post-natal age	Taskforce key term
Electrolyte anomalies (Na, K)	Concentration of serum (meq/L) sodium or potassium below or above the lower and upper limits of normal for gestational and post-natal age	Taskforce key term
Other		
Birth trauma or injury	Neonatal injury associated with delivery	Taskforce key term
	Assessed by physical exam AND birth history	
Allergic conditions*	Infant allergic disorders	Suggested outcome
Autoimmune disorders*	Maternal and infant autoimmune disorders	Suggested outcome
Infant Immunity*	Effect of maternal antibodies on infant responses to active vaccination and/or natural infection Effect of maternal antibodies on infant reactogenicity after active vaccination	Suggested outcome
Gender specific events*	Infant events related to gender	Suggested outcome

Sources of Accepted Definitions: WHO: World Health Organization; BC: Brighton Collaboration; NCS: National Children’s Study; CTCAE: Common Terminology Criteria for Adverse Events (National Institutes of Health); NICHD: National Institute of Child Health and Human Development.

* BC-WHO Consultation consensus term.

a See Brighton definition: Michael Marcy, S., et al., Fever as an adverse event following immunization: case definition and guidelines of data collection, analysis, and presentation. Vaccine, 2004. 22(5–6): p. 551–6.

b See Brighton Definition: Jorch, G., et al., Unexplained sudden death, including sudden infant death syndrome (SIDS), in the first and second years of life: case definition and guidelines for collection, analysis, and presentation of immunization safety data. Vaccine, 2007. 25(31): p. 5707–16.

c See Brighton definition: Bonhoeffer, J., et al., Generalized convulsive seizure as an adverse event following immunization: case definition and guidelines for data collection, analysis, and presentation. Vaccine, 2004. 22(5–6): p. 557–62.

d See Brighton definition: Tapiainen, T., et al., Aseptic meningitis: case definition and guidelines for collection, analysis and presentation of immunization safety data. Vaccine, 2007. 25(31): p. 5793–802.

e See Brighton definition: Sejvar, J.J., et al., Encephalitis, myelitis, and acute disseminated encephalomyelitis (ADEM): case definitions and guidelines for collection, analysis, and presentation of immunization safety data. Vaccine, 2007. 25(31): p. 5771–92.

f See Brighton definition: Wise, R.P., et al., Thrombocytopenia: case definition and guidelines for collection, analysis, and presentation of immunization safety data. Vaccine, 2007. 25(31): p. 5717–24.

g See Brighton definition: idudu, J., et al., Diarrhea: case definition and guidelines for collection, analysis, and presentation of immunization safety data. Vaccine, 2010. 29(5): p. 1053–71.

**Table 3:** Consensus recommendations to further improve safety monitoring of immunization in pregnancy programs.

There is a need for standardized case definitions for an exhaustive set of events. Until these become available, interim definitions should be made available and shared for widest possible use
The phrase “key terms” could be used rather than “adverse events” or “events of special interest” given their respective regulatory implications
Brighton Collaboration provides an open global platform and mechanisms to lead efforts of definition standardization given their previous work on standardizing case definitions at an international level and their expertise on consensus building
An overall evaluation framework should be developed:
a. A public consultation should be implemented for review and feedback on the outputs of the meeting
b. Interim case definitions should be evaluated at least regarding usefulness, applicability, and reduction of inter-rater variability
c. Implementation in simple observational studies (e.g., incidence rate studies) should be pursued and to allow for assessment of applicability and positive predictive values of the definitions and usefulness of terminologies, guidance and tools
d. Case definitions could be incorporated into NIH toxicity tables and tested in clinical trials
Tools should be developed to facilitate implementation at various levels and may include:
a. A data collection tool, such as a case report form/data collection list
b. A glossary of enabling terms
c. Ontologies of the terms, keeping multilingual data collection in mind
d. Disease code mapping for key events should be performed to support case identification
There is a need for guidance(s) for harmonized collection, analysis, and presentation of data in prospective and retrospective data ascertainment
Guidelines should be shared with various stakeholder groups for review and comment
a. This should be done as part of focused stakeholder consultations
b. The Council for International Organizations of Medical Sciences (CIOMS) is considering the establishment of a dedicated working group on immunization in pregnancy which may review and potentially recommend the use of standardized case definitions, guidelines, and tools. The existing CIOMS vaccine pharmacovigilance working group may be able to include the topic with review of the Brighton Collaboration case definitions in their next work plan starting 2017
Population-based health care data sources should be identified and incidence rates of key outcomes should be determined (even if outcome definitions differ from those developed by this group) with a particular focus on LMIC while using advanced databases as benchmarks
The utility of identified databases in LMIC for observational studies including incidence rate, signal substantiation, and hypothesis testing studies should be assessed
Ideally, background rates of key events should be established that are country-specific or site-specific while using common definitions. In practice, this is limited by lack of resources and capacity constraints
Optimal models for conducting post-licensure association studies in LMIC should be assessed including comparison of data collection methods, approaches to meta-analysis and pooling, and performance evaluation of comparative analytic methods to inform interpretation of results from real concerns
Dissemination strategies should be considered
a. The meeting report should be circulated to all participants and for dissemination to their respective institutions
b. Participants should raise awareness of this and subsequent efforts within their institutions and professional networks
c. The two Taskforces should finalize work and publish concepts
d. Brighton Collaboration should make the terms, disease concepts, interim case definitions, guidance, and tools via a dedicated resource platform at its website for immediate use by interested parties
e. Funders should be informed about this ongoing process so that they can inform their investigators about the process and availability of interim case definitions
The Global Advisory Committee on Vaccine Safety (GACVS) is WHO’s principal advisory body on vaccine safety issues. The committee acknowledged the development of global standards for vaccine safety monitoring by the current initiative. Their further endorsement will be critical for acceptance and sustainability of any recommended guidelines and standards
